# Peripheral Ulcerative Keratitis: Pathogenesis, Diagnosis, and Multimodal Management

**DOI:** 10.3390/jcm15031264

**Published:** 2026-02-05

**Authors:** Jose Carlos Guerrero-Acosta, Gustavo Ortiz-Morales, Guillermo Raul Vera-Duarte, Alejandro Navas, Enrique O. Graue-Hernandez, Arturo Ramirez-Miranda

**Affiliations:** 1 Instituto de Oftalmología Fundación Conde de Valenciana IAP, Mexico City 06800, Mexico; a01222949@tec.mx (J.C.G.-A.); gomorales7@gmail.com (G.O.-M.); guillermoveraduarte@gmail.com (G.R.V.-D.); dr.alejandro.navas@gmail.com (A.N.); egraueh@gmail.com (E.O.G.-H.); 2 Tecnologico de Monterrey, School of Medicine and Health Sciences, Monterrey 64710, Mexico

**Keywords:** corneal melt, dry eye disease, immunosuppressive therapy, peripheral ulcerative keratitis, review, systemic vasculitis

## Abstract

Peripheral ulcerative keratitis (PUK) is a sight-threatening corneal disorder characterized by progressive peripheral stromal thinning and ulceration. It often reflects an underlying systemic autoimmune or vasculitic disease and may herald severe morbidity or even mortality. This review offers a comprehensive synthesis of current knowledge on the pathophysiology, clinical presentation, diagnostic approach, and management strategies for PUK, with a special focus on idiopathic forms such as Mooren’s ulcer. We explore the immunological mechanisms driving peripheral corneal destruction, including the roles of complement activation, matrix metalloproteinases, and systemic immune dysregulation. A detailed classification of etiologies, including systemic autoimmune diseases, infectious causes, and iatrogenic forms, is presented, along with recommendations for diagnosis, laboratory workup, and differential diagnosis. Management strategies are reviewed in a stepladder approach, from local anti-collagenase and immunomodulatory therapy to systemic immunosuppressants and biologics, and, when necessary, surgical intervention.

## 1. Introduction

Peripheral ulcerative keratitis (PUK) is an inflammatory disease hallmarked by the triad of crescent-shaped epithelial defects in the peripheral cornea, stromal lysis and an inflammatory infiltrate [1,2]. Historically, its development in patients with rheumatoid arthritis (RA) has signaled the onset of life-threatening systemic vasculitis and a markedly increased risk of mortality; therefore, prompt treatment with systemic immunosuppressive therapy is mandatory [3,4,5].

PUK represents a significant ocular emergency; its progressive nature involves a high risk of corneal melting and perforation, which can lead to blindness [1]. Corneal perforation is the most feared complication as it is associated with poor visual prognosis. Various series report an incidence of 11.5% to 22.7% [4,6]. Systemically, PUK is frequently a manifestation of an underlying and often life-threatening vasculitis or collagen vascular disease and while it usually occurs with long-standing, quiescent RA, it can also occur in up to 60% of patients with granulomatosis with polyangiitis (GPA), often presenting as the first or only sign of severe systemic disease [6,7,8,9]. It can also be the only manifestation of a vasculitis associated with antineutrophil cytoplasmic antibodies (ANCA). [10] Therefore, PUK is a critical finding that warrants a thorough systemic evaluation to find and manage the underlying disease [9].

## 2. Materials and Methods

This article is structured as a narrative review and in compliance with SANRA guidelines. It was conducted using PubMed, Scopus, Web of Science, and Google Scholar from database inception to June 2025. For each database, combinations of free-text terms and Medical Subject Headings (MeSH) were applied, using Boolean operators tailored to the indexing structure of the platform. The following terms were used alone and in combination: “peripheral ulcerative keratitis”, “PUK”, “immune-mediated keratitis”, “corneal melt”, “rheumatoid arthritis ocular complications”, “ANCA-associated vasculitis and eye”, “Mooren’s ulcer”, “collagen vascular disease and cornea”, “ocular vasculitis”, “infectious keratitis”, “ocular surface inflammation”, and “ocular autoimmune disease”.

In addition to the primary database searches, reference lists of key review articles and highly cited original studies were screened to identify additional relevant publications. When duplicate records appeared across databases, they were reconciled and counted as a single study entry.

### Inclusion and Exclusion Criteria

To balance recency of evidence with preservation of foundational knowledge, a dual-search approach was adopted. Priority was given to studies published between 2010 and June 2025, with particular attention to reports addressing current medical management with biologic agents, newer surgical approaches, and updated epidemiological series describing incidence, prevalence, or outcome patterns in PUK. In parallel, pre-2010 publications were intentionally retained when they provided seminal descriptions of pathophysiology, anatomical characterization of the limbal vasculature, or original classification schemes for Mooren’s ulcer and other immune-mediated peripheral keratitis.

Because this is a rare disease, it is expected that high-level evidence such as randomized clinical trials or meta analysis is not readily available. For this reason, eligible sources included case reports, case series, letters to the editor, narrative and systematic reviews, and original observational or interventional studies. Evidence was graded accordingly. Only articles in English, Spanish and French were considered. Conference abstracts without subsequent full-text publication, studies focused primarily on central ulcerative keratitis without clear peripheral involvement, and reports lacking sufficient clinical or methodological detail were excluded. Priority was given to Q1 and Q2 journals; however all articles were assessed for scientific rigor and relevance. The final set of references was assembled to preserve key historical benchmarks while reflecting contemporary diagnostic and management strategies for PUK.

Evidence grading: To help readers interpret the strength of the literature supporting each therapeutic option, we assigned a Level of Evidence to each medication class in Table 1 using the Oxford Centre for Evidence-Based Medicine (OCEBM) framework. Briefly, Level I includes systematic reviews of randomized trials; Level II includes individual randomized controlled trials; Level III includes non-randomized controlled studies and cohort/follow-up studies; Level IV includes case series and case–control studies; and Level V reflects mechanism-based reasoning and expert opinion. When multiple study designs existed for a given intervention, the highest applicable level was reported, while recognizing that evidence in PUK is frequently limited to observational data due to disease rarity.

## 3. Epidemiology

The incidence of PUK is estimated at 0.2–3 cases per million people per year [1,9]. About half of all cases are often related to collagen systemic vascular diseases, mainly RA and GPA [11,12]. In patients with RA, the prevalence is reported at 1.4%, making it the most common systemic etiology for PUK and accounting for up to 34% of all noninfectious cases [13,14]. Across cohorts, it predominantly affects middle-aged individuals with a median age at presentation of 53–69 years [1,6,15]. Female preponderance is most consistent with the demographics of autoimmune disease [16]. Nonetheless, some series report an equal gender balance, noting comparable rates in cohorts from the United States (56%) and Australia [1,6]. Laterality at presentation is of high importance, due to the increased associated all-cause mortality with bilateral presentation, ranging from 14% to 42%, according to current published data [1,6,17,18].

Infectious etiologies account for approximately 20% of cases, while malignancy is a rare but reported association [19].

## 4. Pathophysiology

PUK, by definition, affects the peripheral cornea. This predilection can be explained by unique anatomical and immunological features that distinguish the peripheral cornea from the central cornea. Peripheral limbus favors immune-complex deposition and inflammation, in contrast to the relative immune privilege of the central cornea [9,20].

**Figure 1:**
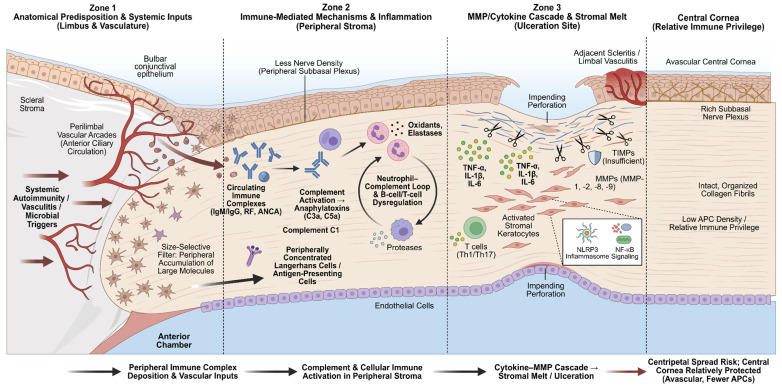
Schematic representation of the pathophysiology of peripheral ulcerative keratitis (PUK) in three zones. Zone 1 illustrates the anatomical predisposition of the peripheral cornea, where perilimbal vascular arcades extend ~0.5 mm into the clear cornea, creating a size-selective filter that traps large immune complexes (IgM, IgG) and shows increased C1 activity compared with the immune-privileged central cornea. Zone 2 depicts immune activation, in which deposited complexes (e.g., rheumatoid factor, ANCA) trigger classical complement activation and anaphylatoxins (C3a, C5a) that recruit neutrophils, eosinophils, and mast cells. Zone 3 shows the terminal effector pathway where pro-inflammatory cytokines (TNF-a, IL-1b, IL-6) drive keratocyte-derived matrix metalloproteinases (MMPs); an imbalance between MMPs and tissue inhibitors of metalloproteinases (TIMPs), sustained by NLRP3 inflammasome and NF-kB signaling, culminates in peripheral stromal melt.

Immune complex–driven complement activation triggers a cytokine–matrix metalloproteinase (MMP) cascade that leads to stromal melt, while additional pathways involving the NOD-, LRR- and pyrin domain-containing protein 3 (NLRP3) inflammasome and increased nuclear factor kappa B (NF-κB) activity perpetuate the inflammatory response [2,21] (Figure 1).

### 4.1. Anatomical and Immunological Predisposition

There are certain features that distinguish the peripheral cornea from the central cornea. Large molecular size limits diffusion into the central cornea, leading to peripheral accumulation near the limbus [22]. Perilimbal vascular arcades extend approximately 0.5 mm into the clear cornea, delivering circulating proteins and leukocytes to the peripheral stroma and epithelium [23]. Biochemical mapping shows about fivefold higher hemolytic C1 activity in the peripheral than the central cornea [22,24]. These anatomical and biochemical features indicate that the limbus acts as a size-selective filter, favoring the deposition of circulating immune complexes, including complement components and IgM, within distal vascular loops [9,20,25]. Antigen-presenting Langerhans cells are concentrated peripherally and thus contribute to inflammatory states; this has been shown in vivo using confocal microscopy (IVCM) in patients with autoimmune diseases [26,27]. The extracellular matrix (ECM) is also different in the peripheral cornea. Stromal lamellae are less uniformly organized than in the center, a configuration thought to be more susceptible to proteolysis during inflammatory states [28]. Likewise, the peripheral corneal epithelium shows a higher MUC4 expression, suggesting distinct glycocalyx signaling at the limbus that may modulate barrier function and cytokine responses [29]. Another important difference lies in regional nerve density, which is less in the periphery, therefore contributing to less sensitivity that may play a role in altered tear dynamics and repair [2,21,30].

### 4.2. Immune-Mediated Mechanisms

In PUK, destruction of the peripheral corneal stroma is a multistep process driven by proinflammatory mediators released from vasculitic limbal vessels [22]. Classical complement activation on immune complexes deposited in the limbal vasculature, including complexes formed by rheumatoid factor and ANCA, initiates the cascade; enrichment of C1 promotes rapid C1qrs assembly on IgM/IgG and generates the anaphylatoxins C3a and C5a [21,25]. These fragments recruit and activate granulocytes at the ulcer margin, where histopathology shows dense neutrophils, eosinophils, and mast cells. Their oxidants, elastases, and collagenases amplify stromal lysis [31]. Macrophages release more proteases, while T-cell dysregulation, including Th1 and Th17 responses with IL-17 signaling, sustains tissue injury [9,21,31].

In systemic autoimmunity, B-cell tolerance defects, such as anti-citrullinated protein antibody responses, increase the immune complex burden that localizes peripherally and perpetuates complement-driven inflammation [31]. Supporting this, serologic studies in PUK associated with RA or GPA document autoantibodies to 54–70 kDa corneal proteins and to epithelial antigens [32].

In the case of Mooren’s ulcer (MU), autoimmunity against specific stromal antigens, such as calgranulin C (S100A12), has been implicated [33,34,35,36]. These mechanisms are discussed in greater detail in Section 7.1.

### 4.3. Role of Matrix Metalloproteinases and Cytokines

Pro-inflammatory cytokines, especially TNF-α, IL-1β, and IL-6, initiate and perpetuate stromal inflammation by activating stromal keratocytes and increasing MMP production [2,22]. This signaling creates a critical imbalance where the activity of collagenolytic MMPs exceeds the restraint of their tissue inhibitors (TIMPs), causing accelerated collagen loss [2,21,22]. Elevated MMP-1 and MMP-8 favor the breakdown of fibrillar collagen, while the gelatinases MMP-2 and MMP-9 target type IV collagen, which undermines the epithelial basement membrane and anterior stroma [37,38]. Keratocytes are a source of MMP-2, whereas the origin of tear MMP-9 remains uncertain. Normal tears show little to no MMP activity, but in severe PUK the tear film contains a prominent 92 kDa gelatinase consistent with MMP-9, along with higher-molecular-weight complexes [21,22,37]. This protease-dominant environment is sustained by key upstream triggers, most notably the NLRP3 inflammasome. Recent histological studies have definitively identified the upregulation of NLRP3, Caspase-1, and IL-1β in the conjunctival tissues of patients with (MU) [39]. While direct histopathological validation in systemic forms of PUK remains to be elucidated (likely due to lack of tissue sampling in patients managed with systemic immunosuppression rather than surgical resection). The NLRP3 finding in MU is mechanistically significant for all forms of PUK because its activation leads to the release of IL-1β, a potent cytokine known to stimulate stromal keratocytes to produce MMP-1 and MMP-2, resulting in accelerated collagenolysis. Furthermore, Zhang et al. [40] reported increased expression of the cGAS-STING signaling pathway in corneal epithelial cells of MU patients, indicating a potential role for cytosolic DNA sensing in initiating the innate immune response. Additionally, overexpression of the succinate receptor GPR91 has been linked to enhanced NF-κB activity in MU tissues, providing a mechanistic link between metabolic stress and sustained inflammation [40].

### 4.4. Systemic Immune Circuits and Local Vascular Pathology

Distinct systemic pathways converge into the same peripheral corneal injury pattern. In ANCA-associated vasculitis, neutrophils are primed through Fc-independent mechanisms and interact with complement (C5a–C5aR axis) to produce a self-amplifying neutrophil-complement loop; ophthalmic series document the same neutrophil-rich, melt-prone phenotype at the limbus [21,31,41,42]. Conjunctival and limbal biopsies in collagen-vascular disease show vasculitis and microangiopathy that align clinically with necrotizing scleritis and facilitate leukocyte trafficking and protease delivery to the corneal periphery [21,31]. Microbial triggers can activate similar downstream cascades via pattern-recognition and neutrophil recruitment; once IL-1/MMP circuits are engaged, the terminal pathway of stromal digestion is shared, irrespective of the inciting antigen [21,37,38,43].

## 5. Etiological Classification

PUK can be produced by a broad spectrum of ocular and systemic disorders, both infectious and non-infectious [21]. Most reviews classify PUK etiologies broadly into systemic, infectious, and surgical/traumatic causes, with a subset related to local dermatologic disease [2]. Among the noninfectious etiologies, systemic autoimmune and vasculitic diseases constitute the largest group, whereas infectious causes contribute to roughly one-fifth of all cases [21,44].

### 5.1. Systemic Autoimmune Disease

Collagen vascular diseases represent approximately 50% of noninfectious PUK cases, positioning the ocular surface as a surrogate marker for life-threatening systemic vasculitis [2,21]. RA remains the most common systemic association, accounting for roughly one-third of noninfectious cases in several cohorts, with bilateral disease reported in approximately half of affected patients [2,21,45,46,47]. PUK usually develops in longstanding RA and often signals the onset of systemic vasculitis [48]. Historical series report a high mortality rate in patients who develop necrotizing scleritis and PUK prior to the widespread use of aggressive systemic immunosuppression [3,4,45].

ANCA-associated small-vessel vasculitides such as GPA present with a distinct clinical timeline; ocular involvement occurs in 50–60% of patients and usually develops in the context of multi-organ disease but can also be the first or sole manifestation [10,49]. The most common manifestations include PUK, uveitis, scleritis and orbital inflammation [45,49].

Other systemic vasculitides and collagen vascular diseases related to the onset of PUK include systemic lupus erythematosus, polyarteritis nodosa, IgA vasculitis, leukocytoclastic vasculitis, relapsing polychondritis, and large-vessel vasculitis [14,17].

Immune complex-mediated disorders such as mixed cryoglobulinemia can also present with PUK. Individual reports describe PUK as the main clinical manifestation of type II cryoglobulinemia and recent studies include it along with dry eye, scleritis, retinal vasculitis, and choroidal ischemia within the ocular spectrum of cryoglobulinemic disease [45,50,51].

Beyond classic vasculitides, PUK has been reported in association with inflammatory bowel disease, psoriasis, ankylosing spondylitis, sarcoidosis and other systemic inflammatory disorders, typically as part of a broader spectrum of ocular surface disease or scleritis rather than as an isolated corneal finding [14,52,53,54].

Finally, a small but clinically important subset of PUK is paraneoplastic, particularly in patients with hematologic or solid malignancies (e.g., leukemia, sebaceous eyelid carcinoma), in whom peripheral corneal melt may be the first clue to an underlying life-threatening neoplasm [19,31].

### 5.2. Infectious PUK

Infectious PUK is the second most common etiologic category as reported in large clinical series. In a prospective Indian cohort, Sharma et al. reported infectious causes in 19.7% of cases [21,44]. Infectious PUK is characterized by a crescentic peripheral stromal ulcer with thinning and an overlying epithelial defect in association with an active stromal infiltrate in which cultures and scrapings confirmed the diagnosis. The causative organism may be bacterial, viral, fungal or parasitic [21]. In the same series bacteria accounted for 73.3% of infectious PUK, most commonly Staphylococcus aureus and coagulase-negative staphylococci, followed by *Pseudomonas aeruginosa*, *Moraxella* spp. and *Streptococcus pneumoniae*; others include *Haemophilus* spp. and *Neisseria gonorrhoeae* [44]. Systemic infections can also present with PUK. In endemic settings one needs to consider tuberculosis in the setting of pediatric PUK, while syphilis has been reported to masquerade as bilateral PUK and to present with perforating PUK [55,56,57].

Viral etiologies which include herpes viruses are another important subgroup. HSV and VZV can produce peripheral or marginal ulcerative keratitis with stromal melting; bilateral herpetic keratitis presenting as PUK has been documented, and herpes-zoster related PUK has also been described in the setting of immunocompromised hosts [2,58,59,60]. Hepatitis B and C have been linked to PUK, the latter in association with medium-sized vessel vasculitis, and the former through mixed cryoglobulinemia and small-vessel vasculitis [50,61,62,63]. MU has also been linked to hepatitis C and helminthic infections [64].

PUK has also been reported as a presenting feature of undiagnosed HIV infection. Reports include cases with severe peripheral melt and corneal perforation [45,65,66].

Fungal keratitis and *Acanthamoeba*, although less common, represent vision-threatening infectious causes of PUK. Care must be taken to rule out infectious disease in patients with high suspicion index before initiating immunosuppressive therapy [2,21,67].

### 5.3. Post-Surgical, Traumatic and Iatrogenic PUK

Post-surgical and traumatic PUK refers to cases where peripheral corneal melt develops after ocular surgery (cataract extraction, pterygium excision, keratoplasty, trabeculectomy and laser in situ keratomileusis (LASIK)) or following ocular trauma, often in patients with an underlying autoimmune predisposition [14,68,69,70]. Corneal trauma-induced immune reaction through altered corneal antigens is thought to be the culprit and can happen weeks to months after uncomplicated surgery [21].

Despite this, the only series (70 patients) to study the incidence of PUK after cataract surgery in RA patients reported no cases of PUK during an 8-week follow-up period [71].

Association between ocular surgery and ocular trauma has been established in an important PUK demographic study that reports 21.5% of patients in their cohort with a history of ocular surgery and 5.88% for ocular trauma [17]. Similarly a study by Sharma et al. [44] report 4% for ocular trauma and 28% for surgery.

Corneal collagen crosslinking (CXL) is a rare post-surgical trigger of PUK, Chanbour et al. retrospectively reported a late-onset sterile PUK in 1.4% (11/711) of eyes undergoing CXL for keratoconus or post-LASIK ectasia, with complications occurring between three months and six years postoperatively [22,72].

Emerging evidence also implicates certain biologic therapies as a cause of PUK; tralokinumab, an IL-13 inhibitor used for atopic dermatitis, has been associated with new-onset PUK in patients without prior rheumatologic history [73] Similarly, miltefosine, the only oral agent approved for leishmaniasis, has been linked to severe ophthalmic toxicity, including cases of peripheral corneal ulceration and MU-like appearance within a recent systematic review [74].

## 6. Clinical Presentation and Differential Diagnosis

PUK is characterized by crescent-shaped peripheral corneal ulceration occurring within 2 mm of the limbus, accompanied by stromal thinning, epithelial breakdown, and an active inflammatory response [2]. The condition can progress rapidly, leading to corneal perforation and irreversible vision loss if not promptly recognized and treated. While PUK can manifest as an isolated ocular pathology, it frequently represents the ocular surface expression of an underlying systemic autoimmune or vasculitic disease [20,75].

Patients with PUK commonly present with intense ocular pain, photophobia, tearing, decreased visual acuity, and a foreign body sensation [14,76]. On slit-lamp biomicroscopy, a peripheral epithelial defect with adjacent stromal infiltration, overhanging edges, and progressive thinning can be observed. Inflammatory signs such as limbal injection and conjunctival hyperemia are almost invariably present. In advanced stages, descemetoceles or frank perforation may form, especially in the absence of timely immunosuppressive therapy [2,5,77].

The clinical presentation can vary significantly depending on the underlying etiology. Idiopathic cases, such as MU, classically present without systemic involvement or scleral inflammation. They may follow a unilateral, indolent course in older individuals or a more aggressive, bilateral pattern in younger patients. In contrast, PUK is associated with collagen vascular diseases, including RA, GPA, polyarteritis nodosa, systemic lupus erythematosus, and relapsing polychondritis [9,78], often manifest alongside scleritis or episcleritis, and may serve as an early marker of systemic vasculitis. In RA, PUK is usually seen in patients with long standing, seropositive disease and may coexist with dry eye and severe ocular surface dysfunction [47,79].

Differentiating PUK from other peripheral corneal disorders is critical. Infectious keratitis, especially due to HSV, presents with hypoesthesia, minimal infiltrate, and typically central or paracentral lesions [80]. Terrien’s marginal degeneration (TMD) causes painless, often bilateral thinning characterized by a gray demarcation line, late-stage lipid deposition, and an intact epithelium [73,81]. Marginal keratitis appears as peripheral infiltrates separated from the limbus by a clear zone (lucid interval); unlike PUK, the epithelium is initially intact, and it is associated with staphylococcal blepharitis [81,82]. Ocular rosacea may mimic PUK with peripheral infiltrates and injection, but is distinguished by lid margin telangiectasia, erythema, and rhinophyma [83]. Finally, Surgically Induced Necrotizing Sclerokeratitis (SINS) must be distinguished in postoperative patients by the presence of adjacent scleral necrosis [68,69].

Correct diagnosis depends on a comprehensive history, systemic review, targeted laboratory workup, and collaboration with rheumatology and infectious disease specialists. Early recognition of these varying presentations is crucial, as the underlying etiology not only guides the diagnostic approach but also significantly influences treatment decisions and long-term prognosis. Accurate differentiation between idiopathic and systemic causes allows for timely systemic evaluation and the initiation of appropriate immunosuppressive therapy, ultimately preserving ocular integrity and reducing the risk of life-threatening complications.

## 7. Diagnostic Workup of PUK

### 7.1. Clinical Features

As previously discussed, PUK is a destructive process mediated by a final common pathway of collagenolytic and proteolytic enzyme degradation. Since PUK may be the presenting sign of a lethal systemic process, early diagnosis and treatment are of paramount importance. The recommended diagnostic approach should consider a thorough history-taking, a comprehensive examination (including ear, nose, mouth, skin, and joints), auxiliary testing, and in a few cases corneal biopsy [84]. During anamnesis, specific events such as previous diagnosis of autoimmune disease, oral or gastric ulcers, nerve palsies, chronic sinusitis, or herpetic keratitis should be questioned.

Diagnosis relies on recognizing the clinical signs detailed in Section 4 and correlating them with a thorough systemic evaluation [84]. However, in ambiguous cases, an ocular tissue biopsy may provide diagnostic information. Performing a biopsy from the adjacent bulbar conjunctiva may support the diagnosis of vasculitis and help exclude infectious and neoplastic etiologies [20].

### 7.2. Systemic Diagnostic Evaluation

Specific systemic manifestations reported by patients or unveiled during systemic examination may point toward the underlying diagnosis, including oral and gastric mucosal alterations, joint pain or deformity, saddle-nose deformity, auricular cartilage changes, malar erythema, and other characteristic skin findings [80]. According to the presence or absence of these signs, laboratory testing for the suspected disease is warranted [17]. Common underlying causes of PUK include RA, GPA, systemic lupus erythematosus, polyarteritis nodosa, relapsing polychondritis, giant cell arteritis, Churg-Strauss syndrome, Behcet’s disease, sarcoidosis, and others. The specific diagnostic criteria for each vasculitic disease are out of the scope of this review; a pertinent multidisciplinary diagnosis in hand with a rheumatologist is desirable. Routine complete blood cell counts and urinalysis are appropriate before and after starting systemic treatment.

## 8. Management

The principles of PUK management rest on four goals: promoting re-epithelialization, halting the keratolysis process, suppressing the underlying ocular and systemic inflammation, and treating the underlying cause [2,3,20,85]. A multidisciplinary approach between rheumatology and ophthalmology using a stepladder approach, beginning with local therapies and escalating to systemic agents based on disease severity and response to treatment, is recommended [3,11,12]. Table 1 summarizes treatment alternatives and dosing regimens for PUK.

### 8.1. Local Therapies

Local therapy for managing symptoms and protecting the ocular surface is the mainstay. This is accomplished with aggressive lubrication using preservative-free artificial tears and ointments (hourly if needed). This helps protect the epithelium, promotes healing, and dilutes inflammatory mediators in the tear film [21]. To halt stromal melting, anti-collagenase therapies can be used, such as doxycycline for its anti-MMP properties, vitamin C supplementation to support collagen synthesis, and N-acetylcysteine as a collagenase inhibitor [12,21,86,87].

The use of topical corticosteroids is controversial and must be paired with systemic control. Extreme caution is required, as these agents can potentiate corneal melting, impair corneal healing, and increase thinning by suppressing collagen synthesis [85,88]. Topical progestins, especially 1% medroxyprogesterone acetate, have been suggested as adjunctive therapy in severe corneal ulceration and stromal thinning because of their glucocorticoid-like anti-inflammatory activity and has been reported to inhibit polymorphonuclear cell-dependent collagenase, which may limit stromal collagen degradation and support collagen repair [89,90]. PUK management reviews mention them as a potential therapy, but available evidence specific to PUK is very limited [14,20,21]. In non-infectious PUK, low-dose steroids (0.1% fluorometholone or loteprednol) two to four times daily can be used to quiet surface inflammation. However, in conditions like RA-associated PUK, some suggest avoiding topical steroids because of reports that they might increase corneal melting [91]. Long-term use is limited to prevent steroid-induced glaucoma and cataract, particularly since these patients may require prolonged courses [21]. Safer alternatives for local immunomodulation include topical cyclosporine (0.05–2%) or tacrolimus (0.1%) [21,92]. Finally, prophylactic topical antibiotics like moxifloxacin 0.5% are recommended to prevent secondary infection of the epithelial defect [2,21].

### 8.2. Systemic Therapy

Systemic therapy is necessary for subduing most of the underlying autoimmune processes. Early and aggressive systemic immunosuppression should be initiated within the first 4 weeks of PUK onset to reduce the risk of perforation, recurrence and preserve vision [5]. Treatment should be staged. Step 1 is corticosteroid induction: oral prednisone is the mainstay for mild–moderate cases, and pulsed IV methylprednisolone is used for severe sight-threatening disease [12,93]; Step 2 is to add immunomodulatory therapy (IMT) as steroid-sparing agents. Methotrexate is the first line for RA-associated PUK. Alternatives include mycophenolate mofetil and azathioprine [5,94,95,96]. For severe necrotizing vasculitis, cyclophosphamide can be used and has been proven effective in refractory cases [79,93]. Step 3 is the use of biologic agents for refractory disease; although their use has dramatically changed outcomes, evidence is largely from case series rather than randomized controlled trials [97]. TNF-α inhibitors such as infliximab or adalimumab are effective in RA-associated PUK, whereas rituximab is highly effective in ANCA-associated vasculitis [49,98,99]. Emerging therapies for multi-refractory cases include Janus kinase (JAK) inhibitors (tofacitinib, baricitinib) and other biologics such as tocilizumab and abatacept [97].

Emerging therapies such as JAK inhibitors have been utilized in refractory cases, though evidence is limited primarily to case series, promising results indicate their possible use as rescue therapy in such cases. Early evidence by Meadow et al. [100] documented partial resolution of a refractory (tofacitinib) PUK case associated with RA. Similarly Calvo-Rio et al. [101] reported rapid remission with baricitinib in a severe case that had progressed to corneal perforation while on tocilizumab. This has been substantiated by recent multicenter data from Sanchez-Bilbao et al. [102,103], who reported an 87.5% complete response rate in 24 patients with refractory inflammatory ocular pathology (including 4 out of 5 (80%) patients with PUK) treated with JAK inhibitors.

Other therapies such as IL-6 inhibitors (tocilizumab) have shown efficacy in some reports but paradoxical worsening in others [104]. Abatacept has demonstrated efficacy in isolated reports, but its role remains controversial; Karampatkis et al. [105] reported the onset of PUK in a patient actively receiving Abatacept and a recent multicenter cohort documented multiple patients previously treated with Abatacept before receiving JAK inhibitors [103]. Finally, agents targeting IL-1 (anakinra) and IL-17 (secukinumab) are theoretically viable due to their systemic efficacy, but their specific role in PUK management remains unstudied.

### 8.3. Surgical Management

Surgical intervention should ideally be performed only after systemic inflammation is controlled medically to reduce the risk of complications [77]. Temporizing and novel measures can be used to stabilize the globe acutely. These include the application of cyanoacrylate glue with a bandage contact lens for perforations smaller than 3 mm, and amniotic membrane transplantation (AMT) to promote healing and reduce inflammation. Novel preparations like freeze-dried amniotic membrane with a spongy layer have shown excellent outcomes [77,106,107,108]. Beyond mechanical support, Amniotic Membrane (AM) transplantation confers biological advantages through its immune-privileged status, mediated by the expression of factors such as HLA-G and Fas ligand, which actively suppress ocular surface inflammation [109]. However, in highly inflamed eyes, the membrane may be rapidly absorbed, providing only transient stability. In such cases, cyanoacrylate glue with a bandage contact lens serves as a more durable alternative for immediate tectonic support in perforations smaller than 3 mm [15].

The use of a subconjunctival dexamethasone implant (Ozurdex) has also been reported as a novel approach for sustained local anti-inflammatory effect [110]. Definitive procedures are reserved for more stable conditions. Conjunctival resection is particularly effective for MU, while lamellar patch grafts are often preferred over tectonic penetrating keratoplasty (PKP) due to a lower risk of rejection. PKP is reserved for large perforations or cases where other techniques have failed [77,111,112,113,114]. Recent case-based evidence supports a scenario-based selection of tectonic procedures for perforated PUK. In a small series of three complex cases with repeated perforations (despite the use of glue), the authors combined intensive systemic immunosuppression with tailored “shaped” approaches: a lamellar sclerocorneal banana graft covering peripheral cornea and sclera in hydrops with multiple small leaks, an eccentric circular peripheral penetrating graft fashioned with two trephines for a large crescentic Mooren-related perforation, and circumferential multilayer stacked amniotic membrane for multiple small peripheral perforations. All eyes maintained globe integrity at 6–36 months of follow-up [115].

**Table 1:** Management Strategies and Treatment Alternatives for Peripheral Ulcerative Keratitis (PUK).

Modality	Therapeutic Agent	Dosing & Regimen	Clinical Remarks, Mechanism & Level of Evidence
**I. Local Therapies (Goal: Lubrication, anti-collagenase, and surface control)**			
**Lubrication** [21]	PF Artificial Tears	Hourly (or as needed)	Dilutes inflammatory mediators; protects epithelium. Level of evidence: V
**Anti-Collagenase** [12,21,86,87]	Doxycycline	100 mg PO BID	Anti-MMP properties to halt stromal melting. Level of evidence: V
	Vitamin C	1–2 g PO QD	Supports collagen synthesis. Level of evidence: V
	N-acetylcysteine	10% topical QID	Collagenase inhibitor. Level of evidence: V
	Medroxyprogesterone Acetate	1% topical QID	Adjunctive for severe ulceration; inhibits PMN-dependent collagenase. **Limited Evidence. Level of evidence: V**
**Anti-Inflammatory** [49,85,88,91,92]	Corticosteroids	0.1% FML or Loteprednol (QID or BID)	**Controversial. May potentiate melting.** Use low potency only to quiet surface inflammation. Level of evidence: V
	Calcineurin Inhibitors	**Cyclosporine A** - 0.05% emulsion Topical BID - 1–2% compounded Topical BID–QID **Tacrolimus:** - 0.03–0.1% drops/ointment: typically BID (up to TID in refractory cases)	Safer alternative for local immunomodulation. Level of evidence: IV
**Prophylaxis** [2,21]	Antibiotics (e.g., Moxifloxacin)	QID or per epithelial defect severity	Prevents secondary infection of epithelial defects. Level of evidence: V (expert opinion, extrapolated from infectious keratitis/epithelial defects)
**II. Systemic Therapy (Goal: Suppress underlying autoimmune process)**			
**Step 1: Induction** [5,12,93]	Oral Prednisone	1–1.5 mg/kg/day (Max 80 mg)	Mainstay for mild-moderate cases. Level of evidence: III
	IV-Methylprednisolone	1 g daily × 3 days	Pulsed therapy for severe, sight-threatening disease. Level of evidence: III
**Step 2: IMT** [5,79,93,94,95,96]	Methotrexate	15–25 mg weekly	First-line for RA-associated PUK. Level of evidence: III
	Mycophenolate Mofetil	1–3 g daily	Steroid-sparing alternative. Level of evidence: IV
	Azathioprine	1–3 mg daily	Steroid-sparing alternative. Level of evidence: III
	Cyclophosphamide	2 mg/kg/day or IV Pulse (0.5–1 g/m^2^ monthly)	Reserved for severe necrotizing vasculitis or refractory cases. Level of evidence: III
**Step 3: Biologics** [49,97,98,99,102]	TNF-α Inhibitors	Infliximab: 5 mg/kg IV at weeks 0, 2, and 6, then q8 weeks (or per rheumatologic protocol); Adalimumab: 40 mg SC every 2 weeks	Effective in RA-associated PUK. Level of evidence: IV
	Rituximab	1000 mg IV on days 1 and 15 (or 375 mg/m^2^ IV weekly × 4)	Highly effective in ANCA-associated vasculitis. Level of evidence: III
	JAK Inhibitors (Rescue therapy)	Tofacitinib 5 mg PO BID (up to 10 mg BID in rheumatology protocols); Baricitinib 2–4 mg PO QD	**Consider only whenrefractoryto conventional treatment.** **Evidence limited to case series. Level of Evidence: IV**
**III. Surgical Management (Goal: Tectonic stability and rehabilitation)**			
**Temporizing** [15,77,106,107,108,109,110]	Cyanoacrylate Glue	With bandage contact lens	For perforations < 3 mm. Level of evidence: IV
	Amniotic Membrane (AMT)	Overlay or graft	Promotes healing; freeze-dried/spongy options show excellent outcomes Level of evidence: III
	Dexamethasone Implant	Subconjunctival (Ozurdex)	Novel approach for sustained local anti-inflammatory effect. Useful for non-compliance or refractory local inflammation. **Evidence limited to case reports**. Level of evidence: V
**Definitive** [77,111,112,113,114]	Conjunctival Resection	Perilimbal excision	Particularly effective for Mooren’s ulcer. Level of evidence: IV
	Lamellar Patch Graft	Partial thickness graft	Preferred over PKP due to lower rejection risk. Level of evidence: IV
	Penetrating Keratoplasty	Full thickness graft	Tectonic. Reserved for large perforations or failure of other techniques. Level of evidence: IV

Summary of current management strategies. Abbreviations: PUK, Peripheral Ulcerative Keratitis; PF, Preservative-Free; MMP, Matrix Metalloproteinase; PMN, Polymorphonuclear; PO, Per os (oral); BID, twice daily; QD, once daily; IV, Intravenous; SC, Subcutaneous; RA, Rheumatoid Arthritis; ANCA, Antineutrophil Cytoplasmic Antibody; IMT, Immunomodulatory Therapy; TNF, Tumor Necrosis Factor; JAK, Janus Kinase; PKP, Penetrating Keratoplasty; FML, Fluorometholone. Evidence for conventional IMT is derived from retrospective cohorts and case series, often combining several agents; data for individual drugs, especially MMF, are more limited (III–IV).

## 9. Special Considerations: Mooren’s Ulcer

MU represents a rare and idiopathic form of PUK, characterized by progressive stromal necrosis of the peripheral cornea in the absence of scleritis or systemic disease [2,64,116]. It is widely regarded as a diagnosis of exclusion, requiring a comprehensive systemic and infectious workup to rule out other potential causes of corneal ulceration [117]. Clinically, it presents with severe pain, photophobia, tearing, and peripheral corneal ulceration with a characteristic overhanging edge [118,119]. The ulcer typically progresses circumferentially and may extend centrally, leading to significant thinning and a high risk of perforation and visual compromise.

The pathogenesis of MU involves complex interactions between genetic predisposition, dysregulated immune responses, and environmental triggers. Despite ongoing research, the precise etiology remains elusive, and MU continues to be a diagnosis of exclusion, posing significant therapeutic challenges. It has been hypothesized that the autoimmune response may be directed against calgranulin C, a neutrophil-derived antigen with high expression in the corneal periphery, which could play a role in initiating or perpetuating stromal inflammation [2,36,120]. Recent evidence suggests that proposed associations with hepatitis C virus and intestinal parasitic infection should be interpreted as possible comorbidities rather than established causal factors [121,122], given the inconsistency and low certainty of available data. The autoimmune response likely arises from molecular mimicry and local immune activation rather than direct infectious etiology.

### 9.1. Clinical Subtypes

Historically, two clinical subtypes have been described based on disease laterality and severity [118,123,124]. The benign or typical variant is generally unilateral, affects older individuals, and tends to follow an indolent course. In contrast, the malignant or atypical variant often presents bilaterally in younger patients and is more aggressive and refractory to conventional therapy [125]. Recent literature relies more in Watson’s classification which stratifies the disease into three distinct clinical phenotypes based on severity and angiographic findings: (1) Unilateral Mooren’s Ulceration, typically affecting patients over 60, characterized by severe pain and rapid progression yet often responsive to medical therapy or surgery; (2) Bilateral Aggressive Mooren’s Ulceration, predominantly affecting younger males (aged 14–40), which carries a high risk of corneal perforation and is frequently refractory to conventional immunosuppression; and (3) Bilateral Indolent Mooren’s Ulceration, commonly seen in middle-aged patients, presenting with a slower, creeping progression that may stabilize with less aggressive intervention [126,127].

### 9.2. Unique Management Aspects

Despite the growing understanding of its immune-mediated nature, a universally accepted therapeutic algorithm for MU remains elusive. Most patients are initially managed with topical corticosteroids and immunomodulators such as cyclosporine A; however, escalation to systemic immunosuppression is frequently required to halt stromal degradation and prevent corneal perforation [118]. Agents such as methotrexate, azathioprine, and cyclophosphamide have been used with variable efficacy [128,129,130,131]. Interferon-α2a has also been reported as a topical immunomodulator in isolated cases [122]. Surgical interventions play a critical role. Conjunctival resection is valuable because it serves a dual role: therapeutically excising perilimbal tissue to remove the source of collagenolytic enzymes, and diagnostically confirming immune etiology to justify aggressive systemic immunosuppression [23,132]. Resection alone may be insufficient in severe MU and it is frequently combined with amniotic membrane or tissue adhesives for tectonic stability. Other procedures include keratoepithelioplasty, lamellar keratoplasty, and AMT, often in combination with immunosuppression, to restore integrity and control inflammation [118,130,133].

Shaped peripheral grafts, such as banana-shaped or eccentric circular tectonic keratoplasty, have also been reported in complex MU with large peripheral perforations and may allow preservation of uninvolved central cornea [115].

A stepwise (“stepladder”) management framework is commonly used in clinical practice, progressing from local supportive and anti-collagenase therapy to systemic immunosuppression, biologic agents, and surgical intervention for advanced or refractory disease. Because high-level comparative evidence is limited for several interventions in PUK, this framework should be viewed as evidence-informed and supplemented by expert consensus and individualized risk–benefit assessment.

Biologic disease-modifying anti-rheumatic drugs (bDMARDs), such as TNF-α inhibitors and anti-CD20 monoclonal antibodies, have shown potential in refractory cases [131]. Additionally, IVCM is a promising tool to monitor subclinical inflammation and guide the tapering of immunosuppressants by assessing dendritic cell density. A recent retrospective cohort demonstrated that topical cyclosporine A 0.05% and tacrolimus 0.03% were associated with rapid resolution of inflammation (within 4–8 weeks), reducing the need for systemic therapy in early-stage disease. These findings support their consideration as effective first-line agents in non-perforated MU. Overall, early diagnosis, prompt immunomodulation, and a multidisciplinary approach are essential to prevent irreversible corneal damage and optimize visual outcomes.

## 10. Prognosis and Long-Term Outcomes

Long-term outcomes in PUK vary substantially across published cohorts and are strongly influenced by the underlying etiology, associated scleral involvement, extent of thinning, and timeliness of systemic disease control. Much of the prognostic literature is derived from retrospective series and heterogeneous case mixes; therefore, reported rates of complications (e.g., perforation, graft failure, recurrence, irregular astigmatism, persistent epithelial defects) should be interpreted cautiously and within the context of study design and patient population.

### 10.1. Systemic Prognosis: PUK as a Marker for Mortality

In systemic autoimmune or vasculitic disease, PUK may signal heightened systemic inflammatory activity and warrants prompt multidisciplinary evaluation. Historical studies—particularly in rheumatoid arthritis associated with necrotizing scleritis—reported increased morbidity and mortality; however, contemporary outcomes may differ in the era of earlier diagnosis and modern immunomodulatory therapy. For this reason, systemic prognostic statements should be individualized and framed in the context of the underlying diagnosis and available evidence [3,4].

### 10.2. The Chronic, Relapsing Nature of PUK

PUK is characterized by a chronic, relapsing–remitting course. Even in patients who initially respond well to therapy, disease flares are common and often occur following tapering or cessation of treatment. Drug-free remission is rare, particularly in MU and other idiopathic forms. Long-term maintenance therapy, whether with topical immunomodulators or systemic immunosuppressants, is frequently necessary to maintain disease control. Clinical vigilance and individualized treatment plans are key to preventing progression and maintaining visual function. Long-term follow-up is essential due to the unpredictable nature of the disease and the high risk of recurrence, especially in bilateral or aggressive variants.

## 11. Future Directions

Future management of PUK should evolve from reactive paradigms toward more predictive, evidence-based care. While systemic immunosuppression and biologic agents have improved disease control, current data remain limited by retrospective designs, heterogeneous protocols, and short follow-up periods. Given the rarity of the disease, future research should prioritize multicenter randomized controlled trials with standardized outcome measures to assess both conventional and emerging therapies, including newer agents targeting the Janus kinase (JAK) and IL-3 pathways.

In the diagnostic realm, although current point-of-care platforms can qualitatively assess MMP-9 activity, there is an unmet need for quantitative tools capable of measuring cytokines and matrix-degrading enzymes across clinically meaningful ranges [134]. Such platforms could potentially allow early detection of inflammatory activity, prior to overt clinical signs, and support tailored adjustments of therapy, offering more objective endpoints for clinical trials.

Advances in surgical techniques, including optimized use of amniotic membrane and customized lamellar grafting, may enhance structural outcomes in complex cases. Looking ahead, technologies such as 3D corneal bioprinting [135], and artificial intelligence (AI) applied to anterior segment OCT and IVCM remain investigational but hold potential to augment future clinical care. These tools may, upon further validation, enable personalized graft designs or standardized imaging biomarkers, such as stromal lysis rate, dendritic cell density, to inform treatment decisions, guide immunosuppression tapering, and define relapse with greater precision.

## 12. Conclusions

Peripheral ulcerative keratitis (PUK) comprises a heterogeneous spectrum of peripheral corneal inflammatory disorders that can threaten ocular integrity and vision and, in systemic autoimmune settings, may reflect underlying vasculitis. Because etiologies range from immune-mediated disease to infection and postoperative or iatrogenic causes, accurate diagnosis—including exclusion of infection—and coordinated systemic evaluation are central to management.

Treatment is commonly approached in a stepwise fashion, combining ocular surface support and anti-collagenase measures with topical and/or systemic immunomodulation tailored to the underlying diagnosis and severity. However, for several interventions the available evidence is derived largely from observational studies and expert consensus. Accordingly, the therapeutic strategies summarized in this review and in Table 1 should be interpreted in the context of the assigned Oxford CEBM evidence levels and individualized to each patient.

Future prospective studies, standardized outcome reporting, and comparative effectiveness research are needed to better define optimal regimens and prognostic factors in PUK, particularly for rare entities such as Mooren’s ulcer and surgically induced disease. Until then, early recognition, multidisciplinary care, and transparent evidence-based (or evidence-informed) treatment planning remain key to improving outcomes.

## Data Availability

No new data were created or analyzed in this study.

## Abbreviations

The following abbreviations are used in this manuscript:

AI	Artificial Intelligence
AM	Amniotic Membrane
AMT	Amniotic Membrane Transplantation
ANCA	Antineutrophil Cytoplasmic Antibody
AS-OCT	Anterior Segment Optical Coherence Tomography
bDMARDs	Biologic Disease-Modifying Anti-Rheumatic Drugs
BID	Twice Daily (Bis in die)
CGAS-STING	Cyclic GMP-AMP Synthase-Stimulator of Interferon Genes
CXL	Corneal Collagen Crosslinking
DNA	Deoxyribonucleic Acid
ECM	Extracellular Matrix
FML	Fluorometholone
GPA	Granulomatosis with Polyangiitis
GPR91	G-Protein Coupled Receptor 91
HIV	Human Immunodeficiency Virus
HLA-G	Human Leukocyte Antigen G
HSV	Herpes Simplex Virus
IgG	Immunoglobulin G
IgM	Immunoglobulin M
IL	Interleukin
IMT	Immunomodulatory Therapy
IV	Intravenous
IVCM	In Vivo Confocal Microscopy
JAK	Janus Kinase
LASIK	Laser In Situ Keratomileusis
MeSH	Medical Subject Headings
MMP	Matrix Metalloproteinase
MU	Mooren’s Ulcer
MUC4	Mucin 4
NF-κB	Nuclear Factor Kappa B
NLRP3	NOD-, LRR- and Pyrin Domain-Containing Protein 3
PF	Preservative-Free
PKP	Penetrating Keratoplasty
PMN	Polymorphonuclear
PO	Orally (Per os)
PUK	Peripheral Ulcerative Keratitis
QD	Once Daily
QID	Four Times Daily
RA	Rheumatoid Arthritis
SC	Subcutaneous
SINS	Surgically Induced Necrotizing Sclerokeratitis
Th1	T Helper Type 1
Th17	T Helper Type 17
TID	Three Times Daily (Ter in die)
TIMPs	Tissue Inhibitors of Metalloproteinases
TMD	Terrien’s Marginal Degeneration
TNF-α	Tumor Necrosis Factor Alpha
VZV	Varicella Zoster Virus
